# Supplementary material for: Functional analysis of CASK transcript variants expressed in human brain
Source: PLoS One. 2021 Jun 16;16(6):e0253223. doi: 10.1371/journal.pone.0253223 (PMC8208546; doi:10.1371/journal.pone.0253223)
Supplement: S1 File — (PDF) [file pone.0253223.s001.pdf]

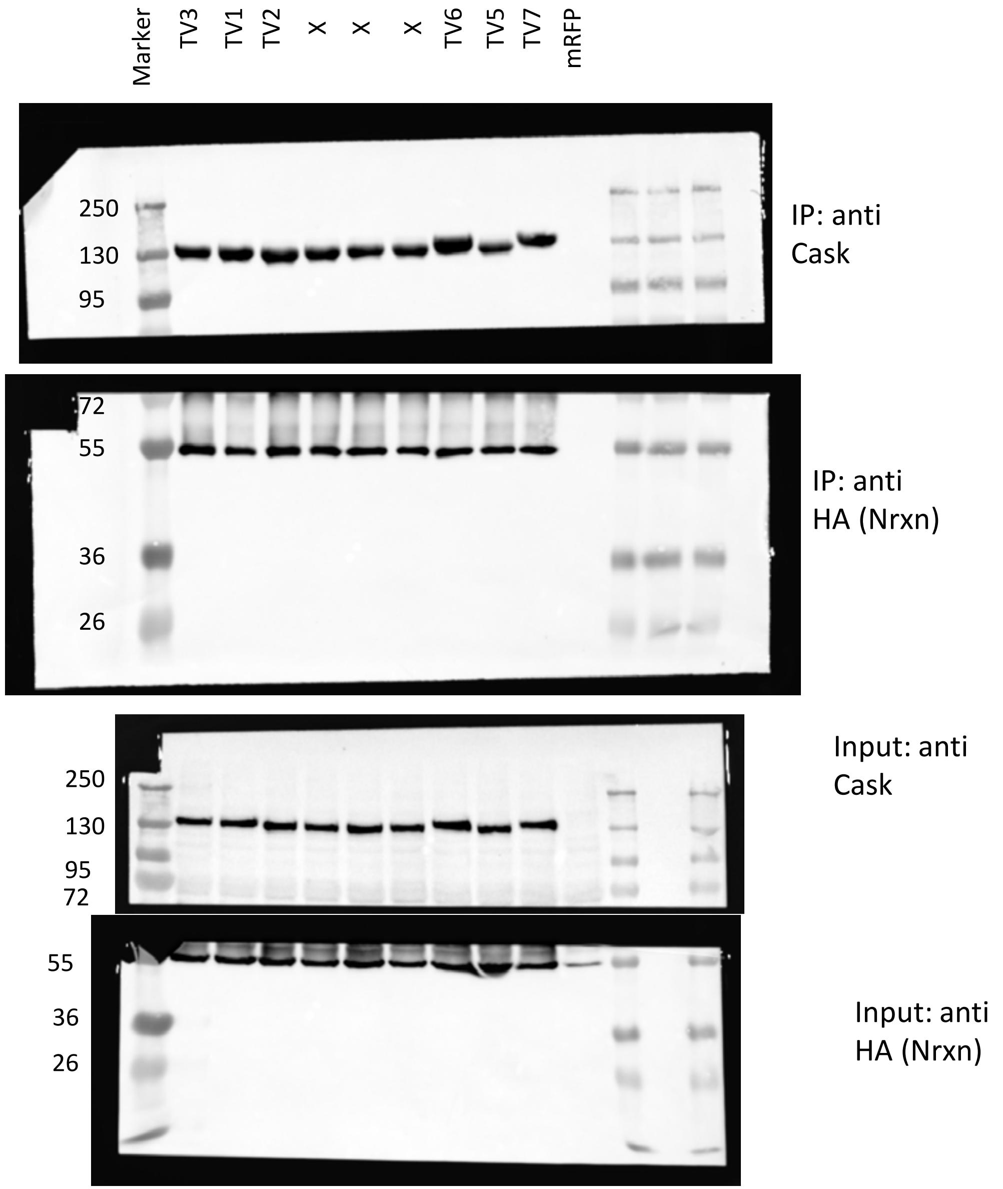

Fig. 2A. Cells transfected with mRFP-tagged CASK variants and HA-tagged Nrxn1

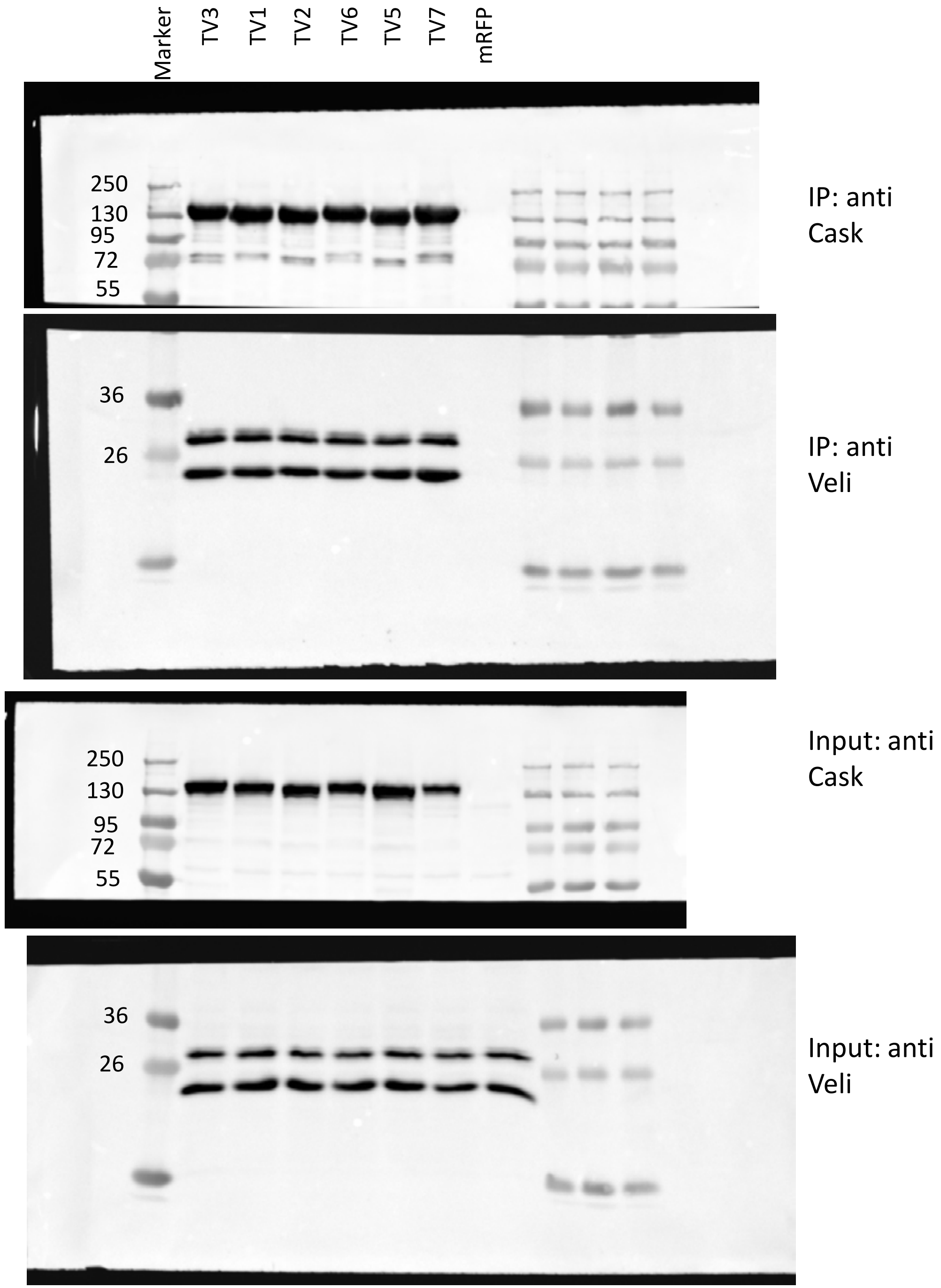

Fig. 3A. Cells transfected with mRFP-tagged CASK variants

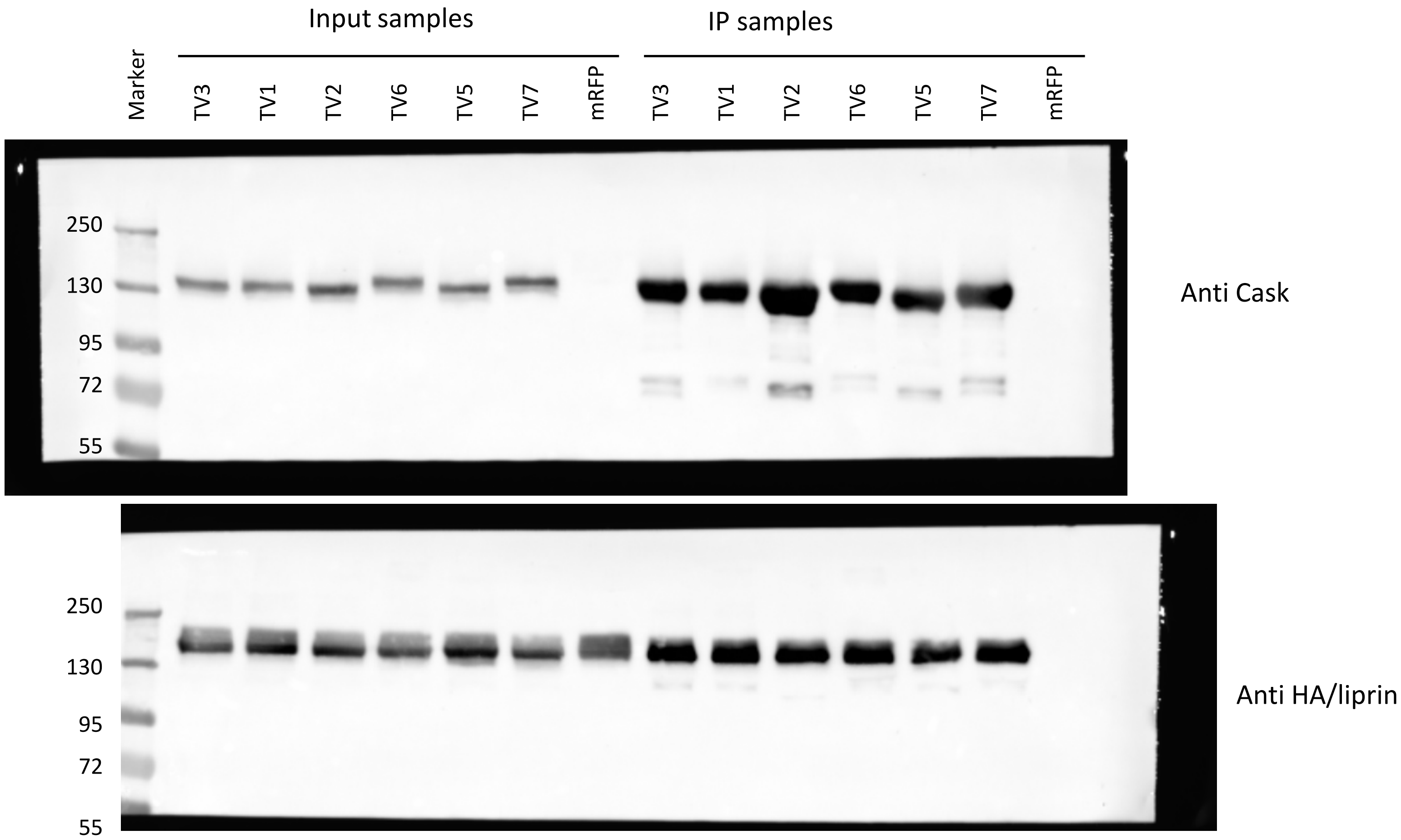

Fig. 4A. Cells transfected with mRFP-tagged CASK variants and HA-tagged liprin

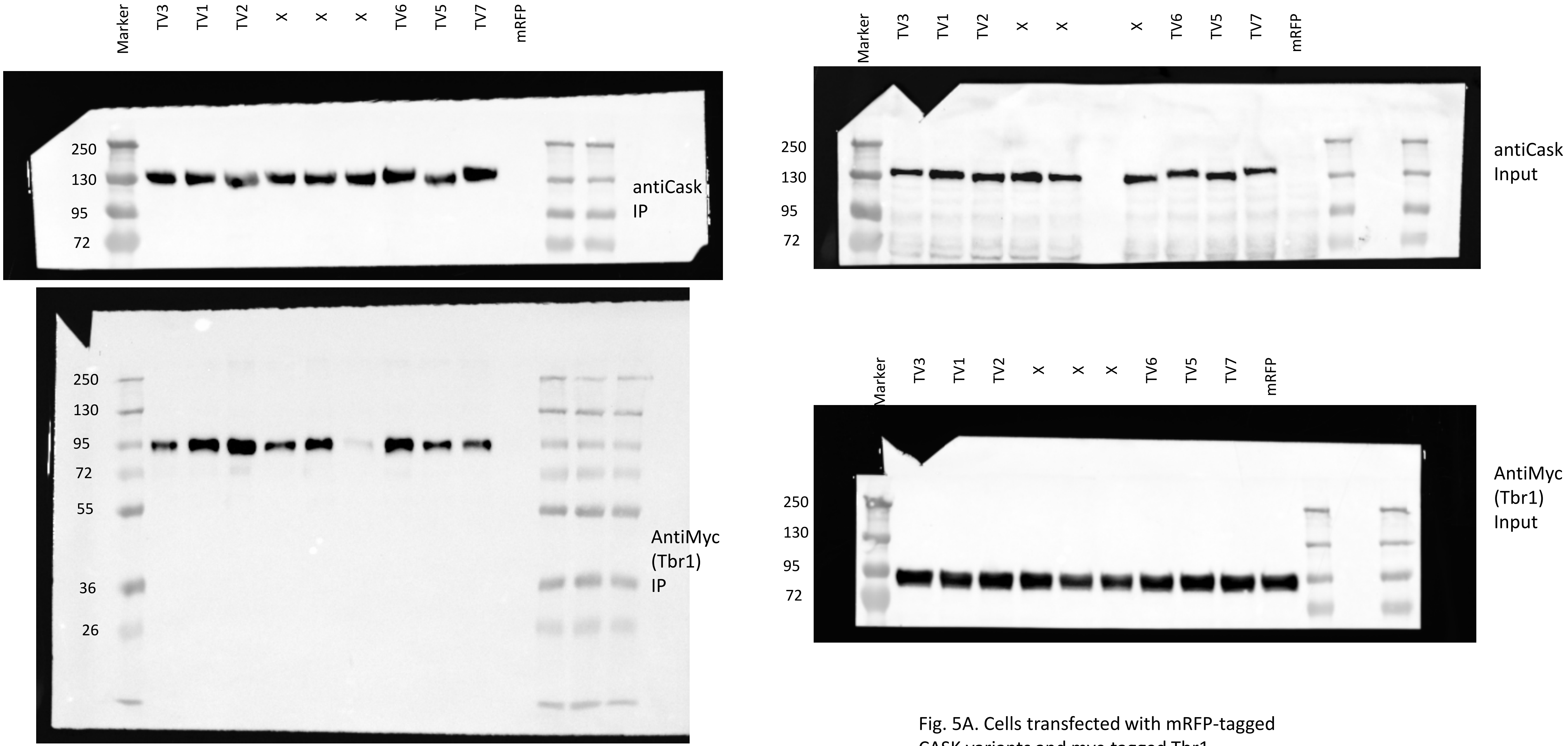

Fig. 5A. Cells transfected with mRFP-tagged CASK variants and myc-tagged Tbr1

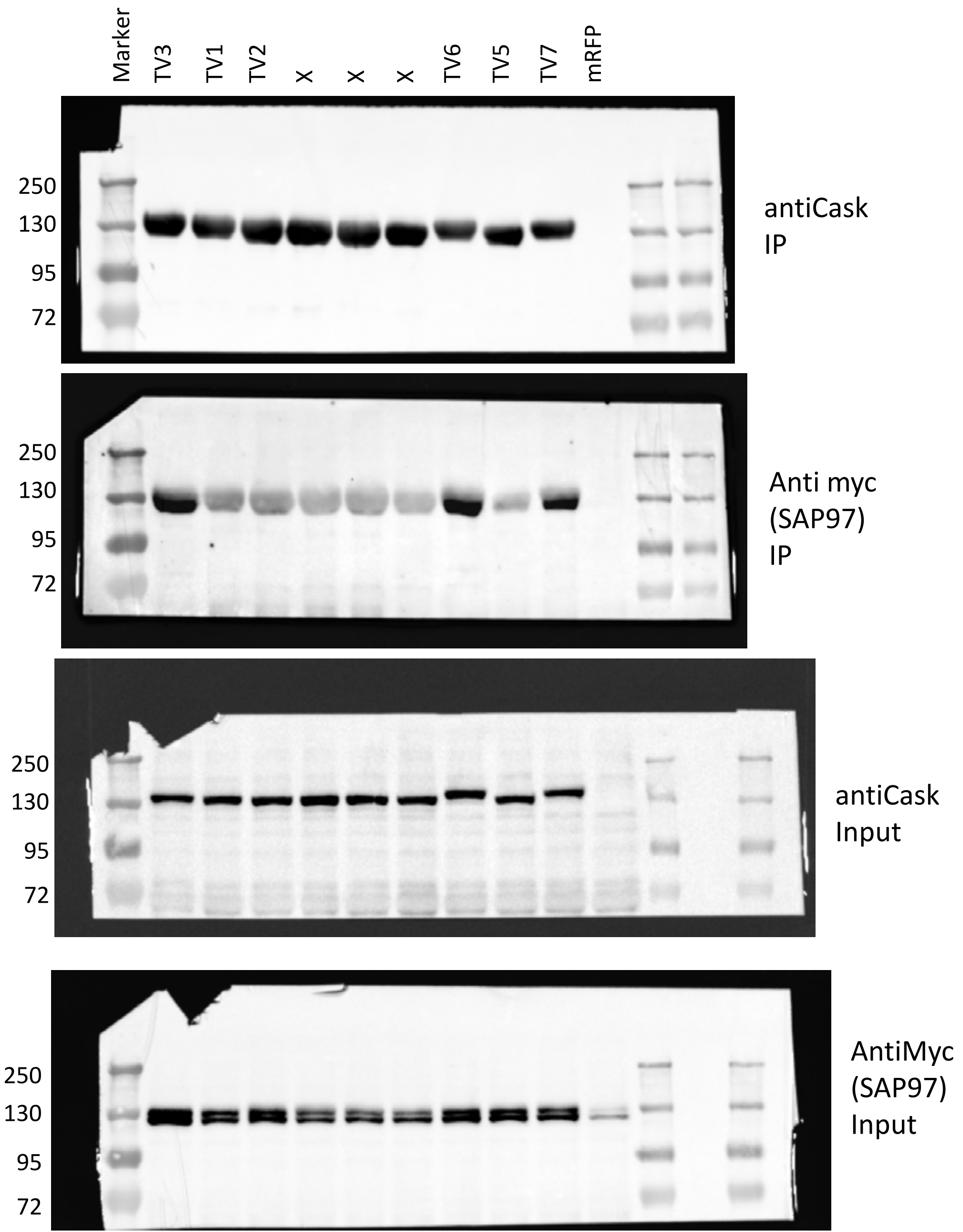

Fig. 7A. Cells transfected with mRFP-tagged CASK variants and myc-tagged SAP97

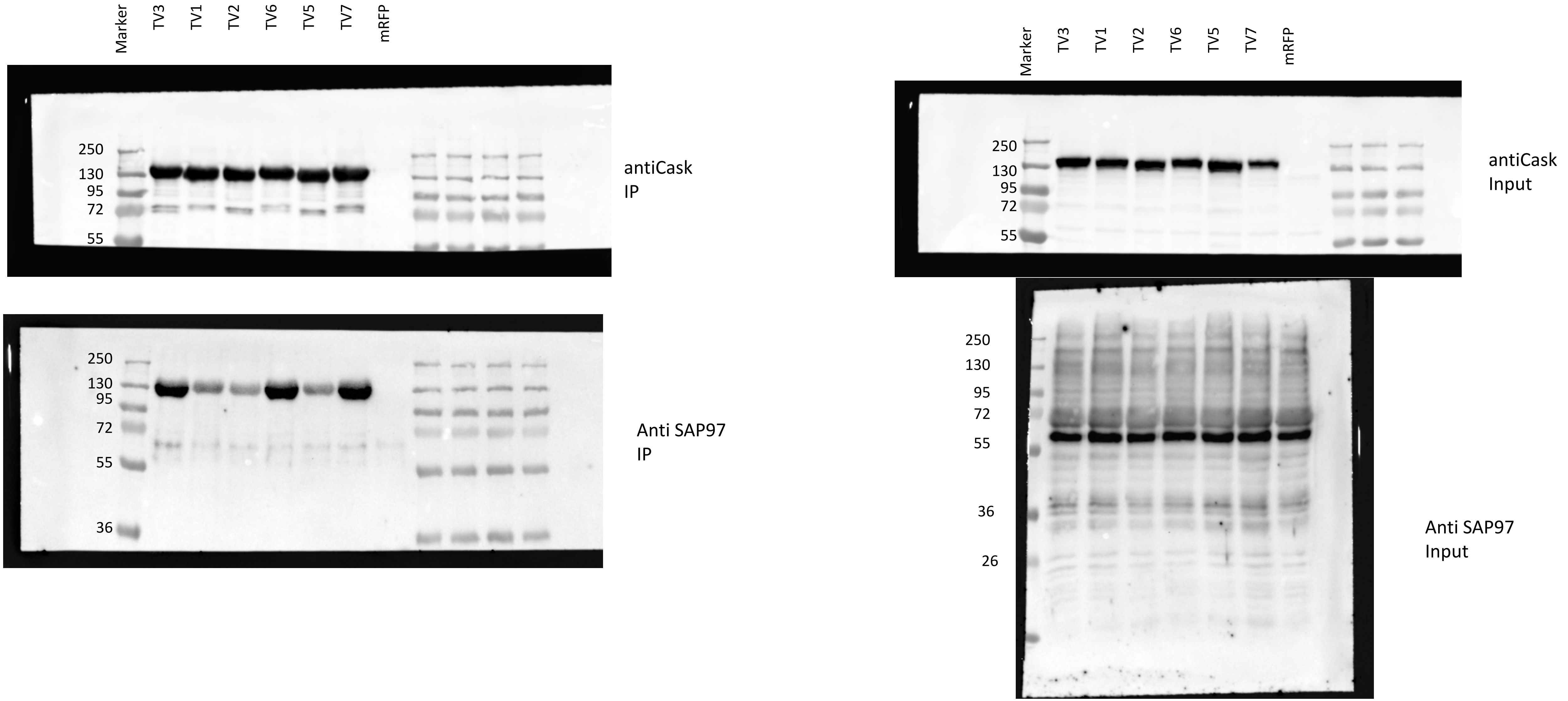

Fig. 8A. Cells transfected with mRFP-tagged CASK variants
