# Supplementary material for: Functional analysis of CASK transcript variants expressed in human brain
Source: PLoS One. 2021 Jun 16;16(6):e0253223. doi: 10.1371/journal.pone.0253223 (PMC8208546; doi:10.1371/journal.pone.0253223)
Supplement: Attachment — Submitted filename: rebuttal.docx [file pone.0253223.s003.docx]

Response to Reviewer’s comments.

Reviewer #2: In general all the questions I asked were answered, however last question about the endogenous Sap97 is now more evident in the sense that also endogenous Veli binds to CASK in all its isoforms. Thus, the discussion should include this finding, why or why not consider endogenous Veli and Sap97in the especificity of the binding to Cask splice variants.

Response: we have now added a more detailed Discussion on the relevance of the two L27 domains of CASK (L27.1 and L27.2), and have detailed why we think binding to Veli is not affected by alternative splicing, whereas binding to SAP97 is affected.
